# Clinical study of lenvatinib in the treatment of hepatitis virus-related hepatocellular carcinoma and antiviral therapy

**DOI:** 10.3389/fphar.2022.1032881

**Published:** 2023-01-10

**Authors:** Xiaomi Li, Jingyan Wang, Xiaoyan Ding, Yawen Xu, Minghua Yu, Hongxiao Wu, Na Deng, Wei Li, Jinglong Chen

**Affiliations:** ^1^ Department of Cancer Center, Beijing Ditan Hospital, Capital Medical University, Beijing, China; ^2^ Department of Interventional Radiology, The Fifth Medical Center, Chinese PLA General Hospital, Beijing, China

**Keywords:** hepatocellular carcinoma, lenvatinib, hepatitis B virus, hepatitis C virus, antiviral therapy

## Abstract

**Background:** Lenvatinib is recommended as a first-line tyrosine kinase inhibitor for advanced hepatocellular carcinoma (HCC) since 2017. The aim of this study was to compare the clinical action of lenvatinib in hepatitis B virus (HBV)-related HCC and hepatitis C virus (HCV)-related HCC.

**Methods:** A continuous cohort of advanced HCC was retrospectively enrolled. And the patients were divided into HBV-related HCC and HCV-related HCC based on previous history of hepatitis virus infection. Then propensity score matching (PSM) was conducted to compare objective response rate (ORR),disease control rate (DCR),progression-free survival (PFS),overall survival (OS) and safety between the two groups.

**Results:** A total of 203 eligible patients were included, with 72 HBV-related HCC and 36 HCV-related HCC after PSM. Both ORR (20.8% vs. 5.6%, P = .0759) and DCR (76.4% vs. 52.8%, P = .0232) were significantly higher in the HBV-related HCC than in the HCV-related HCC. Although no statistical differences in PFS (6.1 months vs. 3.3 months, P = .17) and OS (14.9 months vs. 17.7 months, P = .96) were observed between the two groups, there was a trend of difference in the PFS survival curve. On multivariate regression analysis of PFS, both HBV infection (HR, .54; 95% CI, .31–.95; P = .0332) and antiviral time >5 years (HR, .49; 95% CI, .26–.9; P = .0219) were identified as independent favorable factors, and AFP >200 ng/mL (HR, 1.88; 95% CI, 1.1–3.22; P = .0216) were found to be an independent adverse factor. In addition, compared with HCC who received the first dose of antiviral drugs less than 5 years, the patients who were administered those drugs over 5 years had a significantly favorable PFS (11.27 months vs. 3.87 months, P = .0011). Lenvatinib was well tolerated in all patients and the adverse events (AEs) were similar between the two groups.

**Conclusion:** It seemed that lenvatinib benefited more in HBV-related advanced HCC in delaying disease progression, compared to those with HCV-related advanced HCC.

## Introduction

Liver cancer is the sixth most common cancer worldwide in 2020, with about 906,000 new cases and is the third leading cause of cancer death, with about 830,000 deaths (Sung et al., 2021). Hepatocellular carcinoma (HCC) accounts for 75%–85% as the main histological type (Sung et al., 2021). Viral hepatitis is a major cause of HCC, including hepatitis B virus (HBV) and hepatitis C virus (HCV) infection (Cooke et al., 2019). HBV seroprevalence has continued to decline due to HBV vaccination, and the incidence of HCC has decreased in high-risk countries such as China and the Republic of Korea (Petrick et al., 2020). While vaccine coverage is low in sub-Saharan Africa, HBV-related HCC is still more prevalent and severe (Lemoine et al., 2016). HCV infection occurs mainly in low- and middle-income countries, and although there is no vaccine to prevent HCV infection, direct acting antiviral (DAA) drugs are highly curative and well tolerated (Lanini et al., 2016). Overall, HBV and HCV infection account for 56% and 20% of the global liver cancer deaths, with a huge disease burden (Sung et al., 2021). In clinical practice and guidelines of HCC, the treatment recommendations rely on disease stage and liver function, and they remain the same whatever the reason is HBV or HCV infection.

Early HCC can be potentially curative by resection, thermal ablation, or liver transplantation, and for unresectable patients, local treatments such as trans-arterial chemoembolization (TACE), ablation and radiotherapy can improve patients’ survival (Forner et al., 2018). Moreover, up to 70% patients with HCC are diagnosed at an advanced stage and systemic therapy, such as tyrosine kinase inhibitors (TKIs), is recommended as the first-line regimen (Villanueva, 2019). Sorafenib was the first TKI approved for unresectable HCC, and exploratory analyses of SHARP (Llovet et al., 2008) and Asia-Pacific regions (Cheng et al., 2009) as well as other studies (Peixoto et al., 2014) had shown that sorafenib provided a greater magnitude of benefit in HCV-positive and/or HBV-negative HCC (Bruix et al., 2017; Jackson et al., 2017). For regions with higher HBV infection rates, the benefit of sorafenib was remarkedly smaller (Peixoto et al., 2014) until the advent of another molecular targeted drug. Lenvatinib inhibits vascular endothelial growth factor (VEGF) receptor, platelet-derived growth factor (PDFG) receptor α, fibroblast growth factor (FGF) receptor, and KIT and RET proto-oncogenes (Ikeda et al., 2017). The REFLECT trial demonstrated that lenvatinib was not inferior to sorafenib in overall survival (OS) in the first-line treatment of advanced HCC, with greater improvements in secondary study endpoints such as progression-free survival (PFS), time to progression (TTP), and objective response rate (ORR) (Kudo et al., 2018). The subgroup analysis of this study also demonstrated the benefit of PFS for HBV-related HCC in the lenvatinib group over the sorafenib group (7.3 vs. 3.6 months; HR, .62; 95% CI, .50–.75; *p* < .05) (Kudo et al., 2018). A network meta-analysis showed that lenvatinib was the best mono-therapy for HBV-related advanced HCC in the first-line treatment (Park et al., 2019). Lenvatinib showed better efficacy than sorafenib in a real-world study, and this study highlighted the negative predictive role of HCV on the lenvatinib arm (Rimini et al., 2021).

However, there are no head-to-head studies between different etiologies in HCC treating by lenvatinib, and matching is not strictly performed for comparability. The aim of this study was to compare the clinical action of lenvatinib in HBV-related HCC and HCV-related HCC.

## Methods

### Patients

A continuous cohort of HCC who were treated with mono-lenvatinib at Beijing Ditan Hospital, Capital Medical University from October 2017 to October 2021 were retrospectively collected. Patients over 18 years with hepatitis virus-associated HCC were selected, and required to have at least one measurable lesion by modified Response Evaluation Criteria in Solid Tumors (mRECIST) (Lencioni et al., 2017). In addition, patients included had Child-Pugh grade A/B and Eastern Cooperative Oncology Group performance status (ECOG PS) ≤ 2. Patients who were not on first-line monotherapy, ie, receiving other TKIs or immunotherapy, were excluded. And we removed patients with incomplete baseline data as well as those who were lost to follow-up. Regarding the underlying etiology of hepatitis virus, HBV-related HCC included patients who were positive for HBV surface antigen (HBsAg), HBV core antibody (HBcAb) or HBV e antibody (HBeAb), while patients who were positive for HCV antibody were considered HCV-related HCC, and patients with dual HBV and HCV infection were excluded. Demographic characteristics (etiology and antiviral therapy, age, gender and ECOG PS), baseline clinical data (treatment history, imaging and laboratory parameters) and follow-up data were recorded.

The study conformed to the 1975 Declaration of Helsinki and has been approved by the Ethics Committee of Beijing Ditan Hospital, Capital Medical University. All patients provided written informed consent prior to the study.

### Treatment and assessments

Lenvatinib was administered according to the REFLECT trial (Kudo et al., 2018), and patients weighing ≥60 and <60 kg received initial oral doses of 12 and 8 mg/day, respectively. Dose reductions and interruptions were allowed based on the severity of adverse events (AEs) and tumor progression.

Tumor response was evaluated using mRECIST, and tumor was assessed by contrast computed tomography (CT) or magnetic resonance imaging (MRI). All patients were followed up monthly during the first 6 months of drug treatment and every 3 months after 6 months. The endpoints of this study include ORR, DCR, PFS, OS and safety. ORR was defined as the percentage of complete response (CR) and partial response (PR); DCR was defined as the percentage of CR, PR, and stable disease (SD). PFS is defined as the time interval from initiation of lenvatinib to tumor progression or death, while OS is defined as the time interval from the first dose of lenvatinib to death or last follow-up. Safety was assessed and graded by the Common Terminology Criteria for Adverse Events (CTC-AE, Version 5.0).

### Statistical analyses

All statistical analyses were performed using R software (version 4.0.5). Continuous variables were described using median and range, while categorical variables were expressed as frequency (percentage). In addition, the Mann-Whitney U and Fisher’s exact tests were used to compare continuous and categorical variables, respectively. Kaplan-Meier curves for PFS and OS (median, 95% confidence interval (95%CI)) were performed using the log-rank test to detect the differences between the groups. Propensity score matching (PSM) according to virus species was carried out to control for selection bias, confounding factors included age, gender, PVTT, metastasis and Child-Pugh grade. Univariate and multivariate Cox regression were conducted in matched patients to explore independent factors, and subgroup analysis was to select patients who would like to benefit more. Statistical significance was set at *p* < .05.

## Results

### Patient characteristics

**FIGURE 1:**
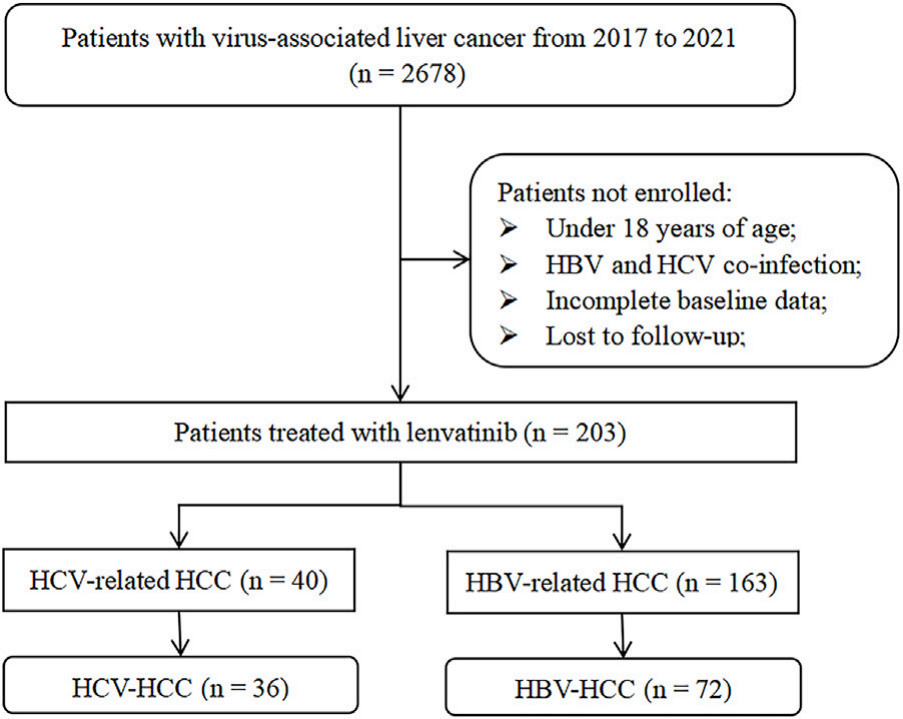
Flowchart of the study.

From October 2017 to October 2021, a total of 203 eligible patients with hepatitis virus-related HCC were treated with mono-lenvatinib, including 163 with HBV-HCC and the remaining 40 with HCV-HCC. After PSM, 72 HBV-HCC and 36 HCV-HCC constituted the study cohort. Figure 1 presents the study cohort selection process. Table 1 summarizes the baseline characteristics of the study population after matching. The differences were eliminated by PSM and balanced and comparable between the two groups. The majority of the PSM populations were males (89.8%), the medium age of the patients was 63.5 years (range: 56.0–69.0 years). Half of the patients had more than 3 tumors, 47 (43.5%) had maximum tumor diameter >5 cm, and the number of patients with PVTT and extrahepatic metastasis was 35 (32.4%) and 51 (47.2%), respectively. Most patients received previous TACE (88.9%), about half received ablation (45.4%), while a few received hepatectomy (14.8%). In addition, 33 (30.6%) patients had Child-Pugh grade B and 72 (66.7%) patients had BCLC stage C.

**TABLE 1:** Baseline characteristics between HBV-HCC and HCV-HCC.

Characteristics	Overall (*n* = 108)	HBV-HCC (*n* = 72)	HCV-HCC (*n* = 36)	P
Age (years)	63.5 [56.0, 69.0]	62.0 [56.8, 69.0]	66.0 [55.8, 69.0]	0.6503
Sex	—	—	—	—
Male	97 (89.8)	65 (90.3)	32 (88.9)	1
Female	11 (10.2)	7 (9.7)	4 (11.1)	—
ECOG (%)	—	—	—	0.6216
PS 0	53 (49.1)	34 (47.2)	19 (52.8)	—
PS 1	45 (41.7)	30 (41.7)	15 (41.7)	—
PS 2	10 (9.3)	8 (11.1)	2 (5.6)	—
Antiviral time (%)	—	—	—	0.9388
≤5 years	79 (73.1)	52 (72.2)	27 (75.0)	—
>5 years	29 (26.9)	20 (27.8)	9 (25.0)	—
Cirrhosis (%)	84 (77.8)	55 (76.4)	29 (80.6)	0.8061
Previous surgery (%)	16 (14.8)	8 (11.1)	8 (22.2)	0.2131
Previous TACE (%)	96 (88.9)	65 (90.3)	31 (86.1)	0.7454
Previous ablation (%)	49 (45.4)	32 (44.4)	17 (47.2)	0.9455
Number (%)	—	—	—	1
≤3	53 (49.1)	35 (48.6)	18 (50.0)	—
>3	55 (50.9)	37 (51.4)	18 (50.0)	—
Size (%)	—	—	—	0.4504
≤5 cm	61 (56.5)	43 (59.7)	18 (50.0)	—
>5 cm	47 (43.5)	29 (40.3)	18 (50.0)	—
PVTT (%)	35 (32.4)	22 (30.6)	13 (36.1)	0.7163
Extrahepatic Metastases (%)	51 (47.2)	34 (47.2)	17 (47.2)	1
Child Pugh (%)	—	—	—	0.8247
Grade A	75 (69.4)	51 (70.8)	24 (66.7)	—
Grade B	33 (30.6)	21 (29.2)	12 (33.3)	—
BCLC (%)	—	—	—	0.8286
Stage B	36 (33.3)	23 (31.9)	13 (36.1)	—
Stage C	72 (66.7)	49 (68.1)	23 (63.9)	—
AFP (%)	—	—	—	0.4081
≤200 ng/mL	77 (71.3)	49 (68.1)	28 (77.8)	—
>200 ng/mL	31 (28.7)	23 (31.9)	8 (22.2)	—

HBV, hepatitis B virus; HCV, hepatitis C virus; HCC, hepatocelluar carcinoma; ECOG PS, eastern cooperative oncology group performance status; TACE, trans-arterial chemoembolization; PVTT, portal vein tumor thrombosis; BCLC, barcelona clinic liver cancer; AFP, alpha-fetoprotein.

### Survival analysis

**FIGURE 2:**
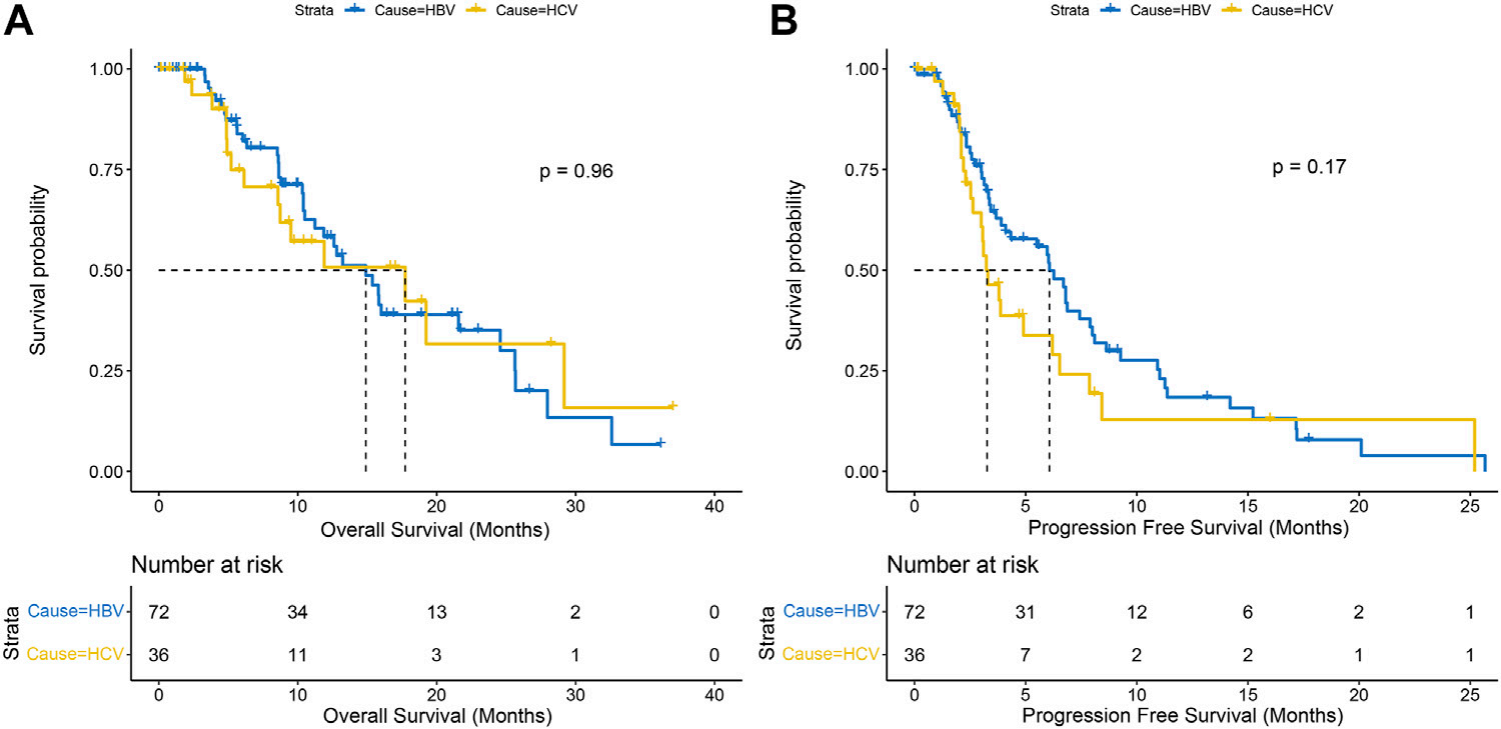
Kaplan-Meier curves of overall survival **(A)** and progression-free survival **(B)** between HBV-HCC and HCV-HCC.

With a median follow-up of 15.6 months, a total of 52 (48.1%) patients died and 76 (70.3%) patients progressed in the matched population, with no significant difference in OS (14.9 months vs. 17.7 months, *p* = .96) and PFS (6.1 months vs. 3.3 months, *p* = .17) between the HBV-HCC and HCV-HCC groups (Figures 2A, B). Although there was no significant difference, we observed a trend of difference in the Kaplan-Meier curves for PFS.

**FIGURE 3:**
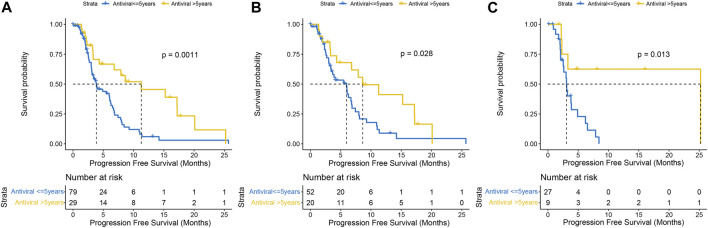
Kaplan-Meier curves of progression-free survival with antiviral therapy earlier than 5 years *versus* less than 5 years in the whole population **(A)** HBV-HCC **(B)** and HCV-HCC **(C)**.

All 108 patients had undergone antiviral therapy, and the anti-HBV treatments were mainly emptecavir, lamivudine, telbivudine, or teenofovir disoproxil fumarate (TDF), while anti-HCV was interferon plus ribavirin before the DAA era, followed by interferon-free direct antiviral therapy. As of last follow-up, more than half (*n* = 42, 58.3%) remained HBV DNA positive in the HBV-related HCC group; whereas most patients (*n* = 29, 80.6%) achieved sustained viral response (SVR) in the HCV-related HCC group. Median PFS was significantly longer in HCCs who had more than 5 years of initial antiviral therapy than in those who had less than 5 years, regardless of virus and drug type (11.27 months vs. 3.87 months, P = .0011) (Figure 3A). In general, patients with HBV infection are treated lifelong, while patients with HCV infection are treated for 3–6 months. Twenty patients in the HBV-related HCC group had antiviral therapy longer than 5 years, while the remaining 52 had less than 5 years, and the former had a significantly better PFS than the latter (8.63 months vs. 5.97 months, *p* = .028) **(** Figure 3B **).** Prior to lenvatinib treatment, antiviral therapy was administered in all HCV-related HCC patients. Nine patients were more than 5 years from their first antiviral treatment and their disease progressed slowly (25.20 months vs. 3.08 months, *p* = .013) compared with 27 patients less than 5 years (Figure 3C).

According to mRECIST, DCR was significantly higher in HBV-related HCC group compared to the HCV-related HCC group (76.4% vs. 52.8%, *P* = .0232). Also, ORR was higher in the HBV-related HCC than in the HCV-related HCC (20.8% vs. 5.6%, P = .0759). Within the HBV-related HCC group, 20.8% (n = 15) subjects achieved PR, 55.6% (n = 40) had SD, and 23.6% (n = 17) had progressive disease (PD). While, in the HCV-related HCC group, 5.6% (n = 2) participants achieved PR, 47.2% (n = 17) had SD, and 47.2% (n = 15) had PD.

### Analysis of factors affecting progression

**FIGURE 4:**
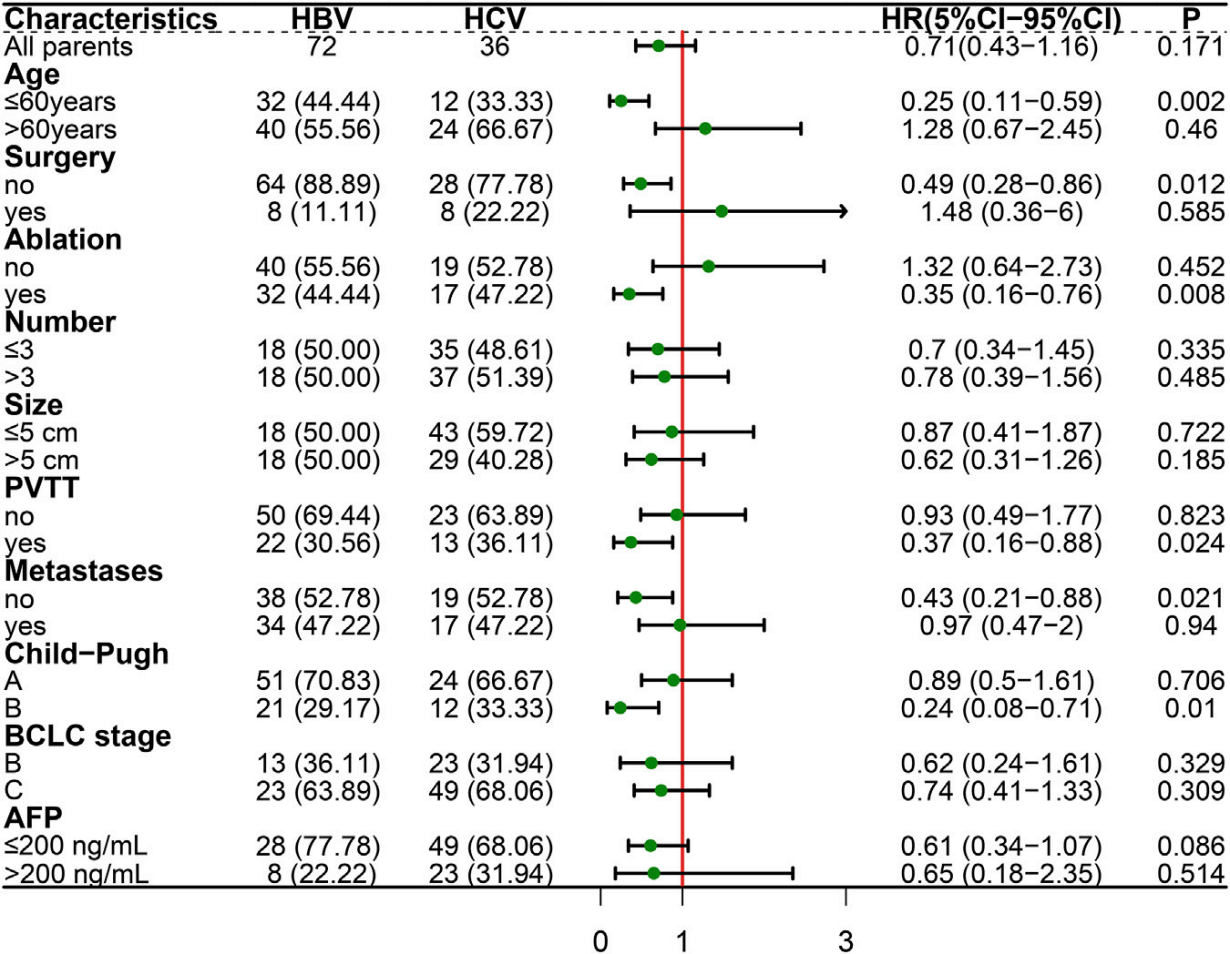
Subgroup analysis of progression-free survival in lenvatinib-treated HCC.

**FIGURE 5:**
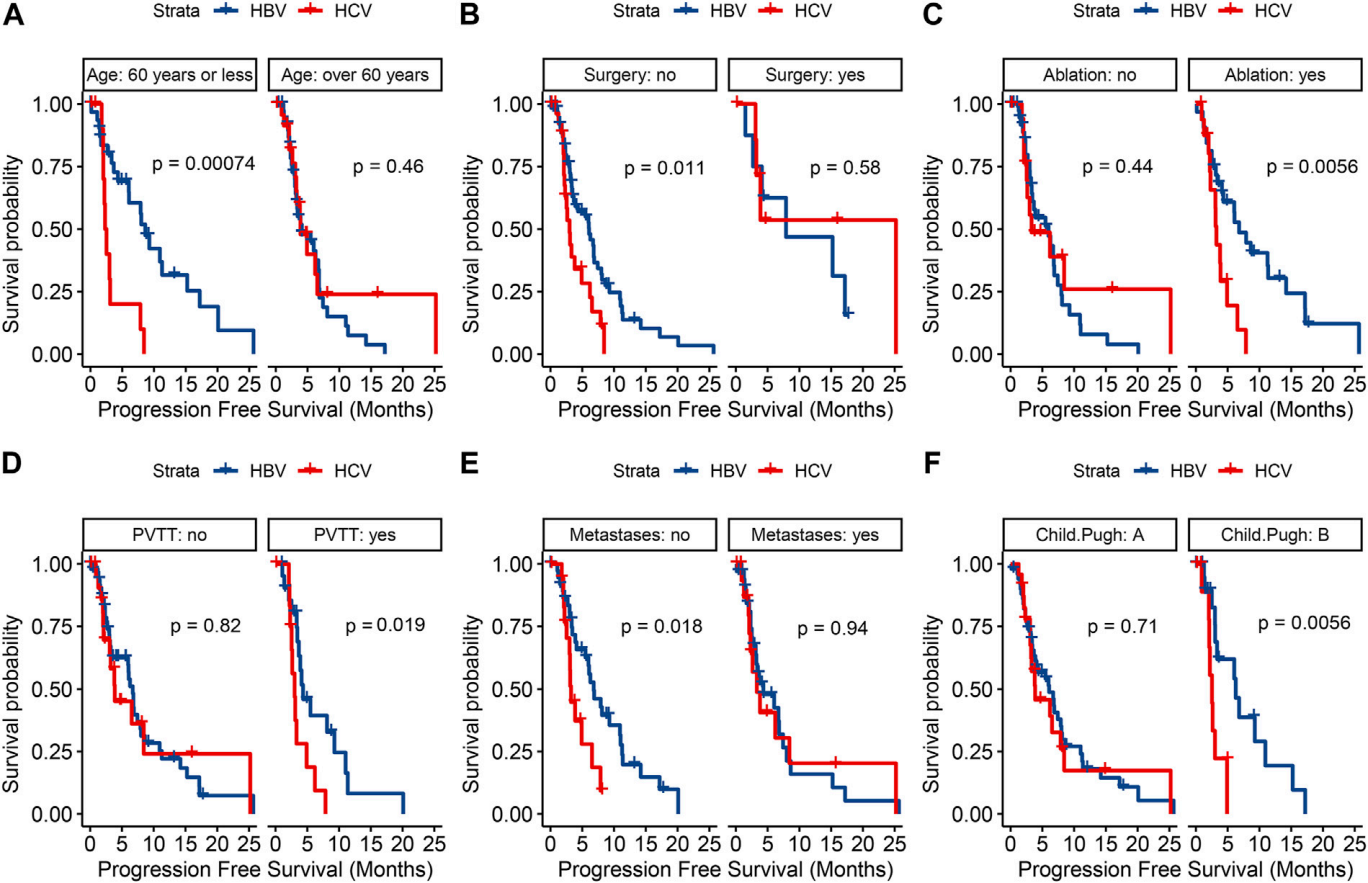
Kaplan-Meier curves for progression-free survival in terms of age **(A)** surgical history **(B)** ablation history **(C)** portal vein tumor thrombus **(D)** extrahepatic metastasis **(E)** and Child-Pugh grade **(F)** between the two groups.

Univariate analysis of PFS showed that age >60 years, HCV infection, antiviral time >5 years, absence of previous surgery, maximum tumor diameter >5 cm, presence of PVTT and alpha-fetoprotein (AFP) > 200 ng/mL were associated with progression in patients treated with lenvatinib (Table 2). Further multivariate analysis, both HBV infection (HR, .54; 95% CI, .31–.95; P = .0332) and antiviral time >5 years (HR, .49; 95% CI, .26–.9; P = .0219) were found to be independent protective factors, and AFP >200 ng/mL (HR, 1.88; 95% CI, 1.1–3.22; P = .0216) was the independent fisk factor for predicting HCC progression. PFS was analyzed in both HBV-HCC and HCV-HCC groups (Figure 4), and the results highlighted HBV-related HCC with age ≤60 years (HR, .25; 95% CI, .11–.59; P = .002), no history of surgery (HR, .49; 95% CI, .28–.86; P = .012), history of ablation (HR, .35; 95% CI, .16–.76; P = .008), presence of PVTT (HR, .37; 95% CI, .16–.88; P = .024), absence of extrahepatic metastases (HR, .43; 95% CI, .21–.88; P = .021), and Child-Pugh grade B (HR, .24; 95% CI, .08–.71; P = .01) had a significantly longer PFS, when compared to HCV-related HCC. And Kaplan-Meier curves of subgroup analysis are shown in Figure 5.

**TABLE 2:** Cox proportional hazards model of prognostic factors for PFS.

Characteristics	Univariate analysis		Multivariate analysis	
	P	HR (95%CI)	P	HR (95%CI)
Age (>60 years vs. ≤ 60 years)	0.157	1.41 (0.88–2.25)	0.4532	1.21 (0.74–1.99)
Sex (male vs. female)	0.864	0.94 (0.45–1.96)	—	—
Cause (HBV vs. HCV)	0.171	0.88 (0.43–1.16)	0.0332	0.54 (0.31–0.95)
Antiviral (>5 years vs. ≤ 5 years)	0.001	0.41 (0.23–0.71)	0.0219	0.49 (0.26–0.9)
Cirrhosis (Yes vs. No)	0.903	1.04 (0.59–1.83)	—	—
Surgery (Yes vs. No)	0.047	0.5 (0.25–0.99)	0.1799	0.59 (0.27–1.28)
TACE (Yes vs. No)	0.904	1.06 (0.39–2.93)	—	—
Ablation (Yes vs. No)	0.629	0.89 (0.57–1.41)	—	—
Number (>3 vs. ≤ 3)	0.462	1.19 (0.75–1.87)	—	—
Size (>5 cm vs. ≤ 5 cm)	0.077	1.51 (0.96–2.39)	0.2572	1.34 (0.81–2.21)
PVTT (Yes vs. No)	0.168	1.4 (0.87–2.26)	0.8855	1.04 (0.63–1.72)
Metastases (Yes vs. No)	0.572	1.14 (0.72–1.81)	—	—
Child-Pugh (B vs. A)	0.231	1.36 (0.82–2.27)	—	—
BCLC (C vs. B)	0.61	1.14 (0.7–1.85)	—	—
AFP (>200 ng/mL vs. ≤ 200 ng/mL)	0.078	1.55 (0.95–2.53)	0.0216	1.88 (1.1–3.22)

PFS, progression-free survival; HR (95%CI), hazard ratio (95% confidence interval); HCV, hepatitis C virus; TACE, trans-arterial chemoembolization; PVTT, portal vein tumor thrombosis; BCLC, barcelona clinic liver cancer; AFP, alpha-fetoprotein.

### Safty

As shown in Table 3, all 108 subjects were analyzed for safety, and the incidence of treatment-related AEs was 81.9% in the HBV-related HCC and 72.2% in the HCV-related HCC. The most common AEs of any grades included hypertension (n = 47, 43.5%), diarrhea (n = 20, 18.5%), fatigue (n = 22, 20.4%), decreased appetite (n = 14, 13.0%), and rash (n = 11, 10.3%), and there were no significant differences between the two groups for any types of AEs. Most of the adverse reactions that occurred were mild to moderate, and few (n = 23, 21.3%) were grade 3. Grade 3 AEs occurred in 16 patients in the HBV-related HCC group, including 6 severe diarrhea, 6 hypertension, 2 proteinuria, 2 hepatic encephalopathy, 2 hyperbilirubinemia, 1 thrombocytopenia, and 1 transaminase elevation; while 7 patients had serious AEs in the HCV-related HCC group, including 3 severe diarrhea, 2 hypertension, 1 proteinuria, 1 hepatic encephalopathy, and 1 hypothyroidism. In total, 7 patients reported severe AEs including 5 upper gastrointestinal bleeding and 2 liver failure, all of which were resolved without sequelae. No significant differences were demonstrated in severe AEs between the two groups. No treatment-related deaths were observed during the study.

**TABLE 3:** Treatment related adverse events.

Adverse events	All (%)	HBV-HCC (%)	HCV-HCC (%)	P
Any grade AEs	85 (78.7)	59 (81.9)	26 (72.2)	0.3607
Hypertension	47 (43.5)	32 (44.4)	15 (41.7)	0.9453
Diarrhea	20 (18.5)	14 (19.4)	6 (16.7)	0.9302
Fatigue	22 (20.4)	13 (18.1)	9 (25.0)	0.5543
Decreased appetite	14 (13.0)	8 (11.1)	6 (16.7)	0.6126
Rash	11 (10.2)	8 (11.1)	3 (8.3)	0.9104
Proteinuria	8 (7.4)	5 (6.9)	3 (8.3)	1
Hypothyroidism	7 (6.5)	5 (6.9)	2 (5.6)	1
Elevated transaminase	5 (4.6)	2 (2.8)	3 (8.3)	0.4182
Nausea/vomiting	4 (3.7)	1 (1.4)	3 (8.3)	0.2073
Hyperbilirubinemia	3 (2.8)	2 (2.8)	1 (2.8)	1
Thrombocytopenia	3 (2.8)	1 (1.4)	2 (5.6)	0.5431
Peripheral edema	3 (2.8)	3 (4.2)	0 (0.0)	0.5278
Abdominal pain	3 (2.8)	3 (4.2)	0 (0.0)	0.5346
Hepatic encephalopathy	3 (2.8)	2 (2.8)	1 (2.8)	1
Grade 3 AEs	23 (21.3)	16 (22.2)	7 (19.4)	0.9338
Diarrhea	9 (8.3)	6 (8.3)	3 (8.3)	1
Hypertension	8 (7.4)	6 (8.3)	2 (5.6)	0.8966
Proteinuria	3 (2.8)	2 (2.8)	1 (2.8)	1
Hepatic encephalopathy	3 (2.8)	2 (2.8)	1 (2.8)	1
Thrombocytopenia	2 (1.9)	1 (1.4)	1 (2.8)	1
Hyperbilirubinemia	2 (1.9)	2 (2.8)	0 (0.0)	0.8008
Elevated transaminase	1 (0.9)	1 (1.4)	0 (0.0)	1
Severe AEs	7 (6.5)	4 (5.6)	3 (8.3)	0.8901
Upper gastrointestinal bleeding	5 (4.6)	3 (4.2)	2 (5.6)	1
Liver failure	2 (1.9)	1 (1.4)	1 (2.8)	1

AE, adverse event.

## Discussion

In this study, we performed a direct comparison between HBV- and HCV-related HCC treated by mono-lenvatinib, and PSM balanced some confounding factors to reduce the bias present in retrospective studies. We observed that both ORR and DCR were higher in the HBV-related HCC than in the HCV-related HCC. Although neither PFS nor OS reached statistical significance after matching, post-matching PFS showed a trend of difference. Moreover, multivariate analysis of PFS showed that HCV-infected HCC had significantly shorter PFS. Univariate analysis of the etiology is not significant, but multivariate analysis is significant might because HBV-related HCC often has a large tumor (Barazani et al., 2007; Sinn et al., 2014), and the independent role of HBV on progression is only revealed when the etiology and tumor size are included in multivariate analysis, eliminating the effect of tumor size. Although HBV-related HCC has higher invasiveness than HCV-related HCC (Cantarini et al., 2006), this study suggested lenvatinib has a protective effect on delaying disease progression in HBV-related HCC. This was confirmed by a real-world analysis that HCV-related etiology is less effective for lenvatinib in HCC (Rimini et al., 2021). In addition, we found that the prolongation effect of antiviral therapy on PFS. Although the duration of anti-HBV is longer than that of anti-HCV, the survival difference was observed in both HBV-related HCC group and HCV-related HCC group.

Although chronic HBV and HCV infection are both the main causes of HCC, there are some differences in the mode of transmission, risk factors and carcinogenic mechanisms (Ng and Wu, 2012). HBV, as a DNA virus, can integrate into the hepatocyte genome, mainly through vertical transmission, and serum DNA level and hepatitis B e antigen (HBe Ag) represent active HBV replication (Chen et al., 2006); while HCV is an RNA virus, mainly through blood transmission, and serum RNA level and viral genotype 1b are its risk factors (Ahmad et al., 2011). In addition, HCC caused by HBV and HCV also differ in clinical manifestations and prognosis (Ng and Wu, 2012), and HBV-infected patients are younger at diagnosis of HCC, and often have larger tumors and PVTT, are more likely to be in advanced stages of the disease, while HCV-induced HCC has poor liver function (Barazani et al., 2007; Sinn et al., 2014). The survival outcomes of the two virus-associated HCC differed in several studies, possibly due to differences in patient baseline characteristics, disease stage and treatment modalities (Cantarini et al., 2006; Barazani et al., 2007; Sinn et al., 2014). Contrast to those results, in our present study, the differences in the prognosis were not detected between the HBV-HCC and HCV-HCC. Also, a meta-analysis showed that there were no differences in OS and disease-free survival (DFS) between the HBV and HCV group (Zhou et al., 2011). The underlying reason in our study maybe that due to the use of PSM, there was no difference in age, tumor size, PVTT and liver function between the above two groups. Subgroup analysis of PFS identified a patient population likely to benefit from lenvatinib treatment. Of note, patients with PVTT and Child-Pugh grade B had a significantly worse prognosis in HCV-infected patients, suggesting lenvatinib monotherapy is poorly effective in these patients and may require systemic therapy replacement. Because HCV-infected patients have worse liver function and patients with Child-Pugh grade B are excluded from the REFLECT trial, more studies are needed to investigate its efficacy and safety (Wong et al., 2011; Sinn et al., 2014).

Most HCC do not show clinical symptoms until they progress to an advanced stage, patients have a poor prognosis, and effective systemic therapy is highly warranted (Forner et al., 2018; Villanueva, 2019). Despite great progress in targeted therapy and immunotherapy in recent years, sorafenib and lenvatinib are currently the standard first-line treatments in clinical practice, while the therapeutic response to targeted drugs is related to viral species. Sorafenib has a survival advantage in HCV-infected patients (Bruix et al., 2017; Jackson et al., 2017), which may be due to the fact that sorafenib can inhibit viral replication and reduce the rate of tumor growth and the deterioration degree of liver function (Himmelsbach et al., 2009; Kolamunnage-Dona et al., 2021). Compared with sorafenib, lenvatinib targets are more concentrated and inhibitory. Indirect comparison showed superior short-term efficacy of lenvatinib, second only to atezolizumab combined with bevacizumab in PFS (Park et al., 2019). HBV infection is associated with favorable prognosis of lenvatinib (Kudo et al., 2018; Park et al., 2019), the mechanism of which is unknown, may result in differential drug response due to different molecular mechanisms of HCC etiology, and may also be associated with lenvatinib modulation of the immune microenvironment (Kato et al., 2019). As an indispensable cornerstone drug for HCC, it is crucial to find reliable biomarkers (such as etiology) and predict their therapeutic response (Doycheva and Thuluvath, 2019).

In addition to the etiology, we observed that serum AFP levels had a role in HCC progression. Serum AFP level is the most commonly used biomarker for evaluating the prognosis of HCC. A multicenter study in Japan found that AFP ≥400 ng/mL was an independent risk factor for death (Tsuchiya et al., 2021). The difference was that the cutoff value of this study was 200 ng/mL, and the study outcome was PFS.

This study had some limitations. First, the sample size of HCV group was small, and the observation period was short, with uncontrollable selection bias; second, we excluded HBV and HCV co-infection, which accounted for a small proportion of patients and was not conducive to analysis.

## Conclusion

Compared with HCV-related HCC, the potential benefit of lenvatinib in delaying progression in patients with HBV-related HCC is more pronounced. However, there is a lack of reliable biomarkers for lenvatinib, and we recommend that viral species should be considered in clinical practice, or stratification by etiology in clinical trials.

## Data Availability

The raw data supporting the conclusions of this article will be made available by the authors, without undue reservation.
